# Hybrid Spider Silk with Inorganic Nanomaterials

**DOI:** 10.3390/nano10091853

**Published:** 2020-09-16

**Authors:** Aleksandra P. Kiseleva, Grigorii O. Kiselev, Valeria O. Nikolaeva, Gulaim Seisenbaeva, Vadim Kessler, Pavel V. Krivoshapkin, Elena F. Krivoshapkina

**Affiliations:** 1 SCAMT Institute, ITMO University, Lomonosova St. 9, 191002 Saint Petersburg, Russia; aleksandra_kiseleva@scamt-itmo.ru (A.P.K.); kiselev@scamt-itmo.ru (G.O.K.); nikolaeva@scamt-itmo.ru (V.O.N.); krivoshapkin@scamt-itmo.ru (P.V.K.); 2 Department of Molecular Sciences, Biocenter, Swedish University of Agricultural Sciences, P.O. Box 7015, SE-75007 Uppsala, Sweden; gulaim.seisenbaeva@slu.se

**Keywords:** spider silk, hybrids, functional materials, inorganic nanoparticles, carbon nanotubes, quantum dots, gold nanoparticles, silver nanoparticles, 3D printing

## Abstract

High-performance functional biomaterials are becoming increasingly requested. Numerous natural and artificial polymers have already demonstrated their ability to serve as a basis for bio-composites. Spider silk offers a unique combination of desirable aspects such as biocompatibility, extraordinary mechanical properties, and tunable biodegradability, which are superior to those of most natural and engineered materials. Modifying spider silk with various inorganic nanomaterials with specific properties has led to the development of the hybrid materials with improved functionality. The purpose of using these inorganic nanomaterials is primarily due to their chemical nature, enhanced by large surface areas and quantum size phenomena. Functional properties of nanoparticles can be implemented to macro-scale components to produce silk-based hybrid materials, while spider silk fibers can serve as a matrix to combine the benefits of the functional components. Therefore, it is not surprising that hybrid materials based on spider silk and inorganic nanomaterials are considered extremely promising for potentially attractive applications in various fields, from optics and photonics to tissue regeneration. This review summarizes and discusses evidence of the use of various kinds of inorganic compounds in spider silk modification intended for a multitude of applications. It also provides an insight into approaches for obtaining hybrid silk-based materials via 3D printing.

## 1. Introduction

Nanoparticle incorporation in the design and development of new materials has been an area of vigorous research in recent decades [1]. Such materials have earned considerable interest in many areas of science and technology due to their remarkable changes in properties, namely mechanical [2], thermal, and magnetic [3], on transfer into the nanoscale. On the other hand, natural polymers often exhibit exceptional mechanical performance due to their highly ordered molecular structure [4], and show high biological stability and controllable bio-degradability due to their natural origin [5]. In recent years, use of the natural fibers for assembling innovative hybrid materials has expanded drastically [6]. Recent advances in biotechnology and composite science, such as different synthesis methods and characterization techniques, and various modes and mechanisms of material degradation, have opened up astounding prospects for making innovative functional materials based on natural biopolymers [7,8]. Spider silk is a distinct class of natural biopolymers, which is given extensive consideration because of its complex progressive structure and specific pattern of chemical and physical properties [9,10]. As it is a protein formed in glands in a spider’s body, spider silk can be used for numerous purposes such as spider transportation, courtship, shelter, and prey trapping. As indicated by ongoing research, the set of mechanical properties (such as elongation, elasticity, toughness, and Young’s modulus) and high versatility of spider silk at moderately low fiber diameters fundamentally surpass those of the silkworm silk and make it a suitable material for various applications in the fields of biomedicine and materials engineering [9,11,12,13]. Apart from its mechanical properties, the high biocompatibility and controlled biodegradability of spider silk merit special mention [14,15,16]. The combination of these unique features therefore makes spider silk a very appealing fiber for applications in medicine, protective materials, and textiles [17], since it is possible to produce a structurally stable and compact biomaterial resistant to degradation under human physiological pH.

Spiders make exceptionally developed silk fibers with a colossal functional capability of the material through a controlled assembly of proteins named spidroins into task-specific fibers (up to 7 different types) [18]. Certain outcomes have been accomplished in studying the process of natural production of this complexly arranged fiber in the silk-producing organs of the spider [19,20]. During the processing of spider silk under changes in temperature, pressure, and pH in a narrow spinning duct, the spidroins arrange into an ordered nanocomposite fiber with a complex microstructure [21,22]. This procedure shows the development of hierarchically organized stimuli-responsive materials based on natural structures that can respond to changes in pH and ion concentration on the molecular level [23]. This information has expanded the interest in spider silk as a basis for forming a variety of biomaterials for different practical applications [23,24]. Moreover, as the hierarchical arrangement of spider silk fibers is possible because of protein intra-and intermolecular interactions [25,26], it implies that these microarchitectures can be used to incorporate inorganic segments into silk filaments to deliver additional mineral and functional loads to biopolymer hybrids. Molecular mechanisms in interactions of spider silk with inorganic nanomaterials are less investigated. To the best of our knowledge, the first example of molecular model studies relying on single crystal structure data on complexes of polyoxometallates with oligoglycine peptides has recently been published only recently [27].

Spider silk structural features and its implementation as an organic scaffold for hybrid materials production have been already discussed by the authors in a related work [28]. However, previous review was namely focused on spider dragline silk and its comparison with silkworm silk, while in this review a variety of silks was described in detail in accordance with arthropods’ infraorders (Araneomorphae, Mygalomorphae). Furthermore, the resulted spider silk-based hybrids were divided into groups based on the incorporated inorganic component (metal nanoparticles, metal oxide nanoparticles, inorganic salts, carbon nanomaterials) with their subsequent characterization, paying major attention to new functional properties that can emerge in such composites. The authors also highlighted the perspectives of 3D printing as a fabrication method and noted its potential features in addition to previously described synthetic approaches [28].

Thus, currently there emerges a window of opportunity to modify silk with various nano scaled inorganic components, namely metal and metal oxide nanoparticles, inorganic salts and carbon nanomaterials [29]. In this context, this review summarizes recent progress in the construction and use of hybrid materials based on natural or recombinant spider silk and inorganic nanoparticles. It is especially worth noting that although silkworms are more domesticated and their silk has been used by industry for millennia to produce textiles, a variety of fiber types combined with superior mechanical properties make spider silk a much more interesting material for research and development. In this regard, this review focuses solely on advances of functional materials based on spider silk, which distinguishes it from several works dedicated to silkworm silk fibers [30].

## 2. Spider Silks Characterization and Properties 

Spiders are a widespread order of arthropods, inhabiting various ecosystems. Currently, 35,000 of various spider species are known. Even though most spider-rich areas are those with abundant vegetation and enhanced humidity, these animals still may be found in different climatic zones from the polar regions and high mountains to dry steppes and hot deserts. It is clear that spiders owe this evolutionary success to their ability to produce different types of silk for a variety of needs. Silk threads are characterized by high tensile strength comparable to Kevlar and enhanced flexibility that makes spider silk unique among both natural and artificial materials [24]. Spider silk is resistant to environmental conditions, biocompatible and biodegradable, which is due to the special silk structure [10]. 

**Figure 1:**
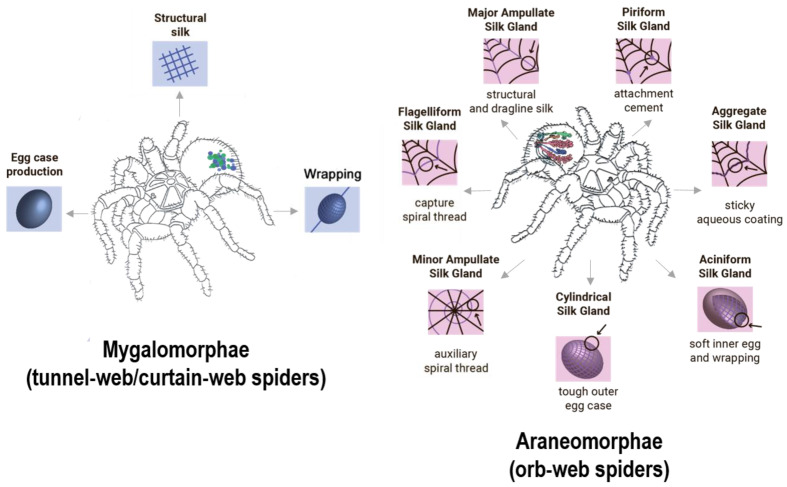
Schematic representation of morphological differences in spider glands between Araneomorphae and Mygalomorphae species with corresponding silk fiber functions.

During the evolution process, a successive transformation of complex mechanisms for creating and using spider web occurred [31]. Morphological differences in the spinning glands construction played crucial role in the formation of two main infraorders of spiders: Araneomorphae and Mygalomorphae (Figure 1).

### 2.1. Araneomorphae Silk and Its Chemical Composition

Representatives of Araneomorphae—*Nephilia clavines* [32], *Araneus diadematus* [33], *Argiope aurantia* [34], are widely known and well-studied. The spinning apparatus of Araneomorphae spiders has a complex structure: in their abdominal cavity there are up to 7 different glands producing silk fibers with different mechanical characteristics depending on the needs of the spider [18].

The variety of fibers mechanical characteristics and morphological differences between glands correlate directly with their functions in the daily life of a spider (Table 1). Each gland secretes a set of proteins of specific composition. There is a general convention according to which the name of each type of protein is selected: the first part of the word indicates the type of the gland, where the proteins were secreted, followed by Sp for spidroin and ends with the serial number, which marks different members of the family (e.g., MaSp1 for major ampullate spidroin 1).

The molecular composition of spidroins is formed by repetitive amino acid units forming the base monomer. Monomers, in turn, multiply and create thousands of building blocks that make up a fiber:

(1) The GPGXX unit (X = Q, G, Y), which forms a β-turn spiral structure, is responsible for the exceptional elastic properties of flagelliform silk [35].
(2) An or GAn are alanine-rich motifs containing 6–9 alanine amino acids that form crystalline β-sheets, exceptionally important for tensile strength [36,37]. Dragline silk is especially rich in alanine motifs, which are the main reason for high tensile strength, being the key feature that characterizes draglines [38]. Fragelliform silk in contrast to draglines, does not contain as many alanine units.
(3) GGX unit (X = Y, L, Q) is rich in glycine, which is known by forming 310-helical units and connecting different crystalline regions (stacks of β-sheets). This structure is responsible for the elastic properties of draglines and flagelliform silks [39].
(4) Spacers: contain charged groups and split repetitive peptide units into clusters [40].
(5) NR: non-repetitive regions at the amino- and carboxyl-ends of proteins [41].

The major and minor spidroins (MaSp1 and 2; MiSp1 and 2) are structurally similar and are produced in ampullate glands. These proteins are both mechanically strong because the (GA)n/An β-sheet module, which is presented in at least one of named proteins [42]. The elasticity of the fiber is provided by the (GGX)n and (GPGXX)n modules mainly composed of less ordered building blocks 31-helices and β-turns [43]. MaSps are the main component in the well-studied dragline silk. This silk is best characterized by its mechanical properties and outperforms most human-made fiber-like structures (Table 2).

Flagelliform gland is the source of Flag silk, which features more than 200% elasticity through multiple repetitions of the GPGXX module [44]. Such ability is needed for spiders to dissipate the impact energy of prey, when it crashes the spider web.

Tubuliform silk is produced in Cylindrical (or Tubiliform) gland by adult female spiders to protect spider eggs in the egg case. The protein composition in this type of silk is different from those described above. Repetitive units of TuSp are mainly composed of alanine and serine, which form the new motifs Sn, (SX)n, and GX (X = Q, N, I, L, A, V, Y, F, D) [45].

Pyriform silk is used by spiders to produce disks for silk attachment to different substrates (anchor points for the dragline, the frame of a web, to attach egg cases to a surface). PySp is also composed of repeated amino acids, which are formed by two exclusive repetitive motifs (a glutamine-pair-rich (QQ) sequence such as QQSSVA, and domains of alternating prolines (PXPXP) [46]).

When it considers prey-wrapping or forming inner layer of eggs, spiders use aciniform silk. Aciniform threads are composed of approximately 200-amino acids, but do not demonstrate sequence similarity to most commonly described spidroins. According to Raman spectromicroscopy, aciniform fiber composition is dominated by moderately oriented 30% β-sheets and ~50% α-helices [47].

Aggregate or Glue silk is produced solely by ecribellate spiders, and it serves as droplets on specific structures in their prey-capture mechanisms. These droplets contain various proteins, among which there are two prior glycoproteins aggregated spider glue 1 and 2 (ASG-1 and -2). The stickiness of the glue droplets is connected with the type of surface the glue attaches to [48].

**Table 1:** Mechanical properties of natural spider silk fibers (Araneomorphae).

Spinning Gland	Function	Protein	Tensile Strength (MPa)	Young’s Modulus (GPa)	Elongation (%)	Ref.
Major ampullate gland	Dragline; frame of the web	MaSp1; MaSp2	1200 ± 200	3.4–11.5	25–35	[49]
Minor ampullate gland	Auxiliary spiral threads	MiSp	900 ± 50	3.0 ± 0.6	5	[50]
Fragelliform gland	Core fiber for prey capture	Flag	800 ± 100	0.012–0.08	≥200	[51]
Cylindrical gland	Outer egg sac	TuSp	400 ± 50	8.7 ± 0.9	5–20	[52]
Pyriform gland	Attachment element	PySp	100 ± 40	0.2 ± 0.1	50–80	[53]
Aciniform gland	Inner egg sac; prey wrapping	AcSp	600 ± 50	10.4 ± 1.4	80	[47]
Aggregate gland	Aqueous coating	AgSp	800 ± 200	1.0 ± 0.1	50–100	[54]

**Table 2:** Mechanical properties of natural and human-made fibers.

Material	Tensile Strength (MPa)	Young’s Modulus (GPa)	Elongation (%)
Dragline silk	140–1600	3.4–11.5	16–350
Bombyx mori (silk)	500–600	9.6 ± 0.6	70
Elastin	2	0.001	1.6
Kevlar	3600	130	60
Nylon 66	750–950	2–3.6	80
High tensile steel	1650	200 ± 10	6
Carbon fiber	4000	300	25

### 2.2. Mygalomorphae Silk and Its Chemical Composition

Spiders belonging to the Mygalomorph group have been less studied, since representatives of this infraorder retained their ancestral characters. 3000 species of Mygalomorphae spiders have been described, among which typical representatives are tarantulas (*Theraphosa blondi*), funnel web spiders (*Hexathele hochstetteri*) and curtain web spiders (*Linothele fallax*, *Euagrus*). Spiders of this group are powerful predators, since they are equipped with large fangs and venomous chelicerae. Mygalomorphs do not need silk to catch prey because of these adaptations. Therefore, they do not build orb-webs, using silk instead to build burrows, produce egg sacs, extend their sensor abilities and wrap their prey [55]. In case of this group, silk is produced in simplified globular undifferentiated spinning glands [56].

Despite the simplified structure of the glands, Mygalomorphs are still of interest for studying the natural web. Due to the specific features of their lifestyle, spiders of this group lack any specialized applications for their silks that would generate highly engineered threads. The absence of silk threads variety is compensated by the large size of the spiders themselves. Mygalomorphae possess an enlarged abdomen [57], due to which they can produce enough cobwebs, for example, to create a large dwelling. Based on observations of adult spiders from the family Dipluridae (*Linothele fallax*) at the local insectarium in SCAMT Institute, it was found that one adult spider can secret up to 10 mg of cobwebs per week. This amount of cobweb is enough to perform various kinds of experiments. In addition, Mygalomorphs are known for their longevity, as spiders of this group can live up to 25 years.

Chemical composition of spidroins within Mygalomorphae species still demonstrates a repetitive nature, but the length of repeats is much longer in comparison with Araneomorphae spiders [58]. Several spidroins of this group have been indicated with the example of *A. juruensis* spider. Repetitive units in Spidroin 1 are composed of 180–190 amino acids approximately with a predominance of serine and alanine-containing sequences. There were also indicated small inclusions of threonine, which was not a typical amino acid found in spider silk [31]. Composition of Spidroin 2 has also been identified. It turned to be somewhat similar to this of Bombyx Mori fibroin. The most frequent amino acids in Spidroin 2 structure are serine, alanine, and glycine, representing ~75% of the fiber protein composition [59]. (GA)n, a few of the An and GPGXX motifs were indicated in repetitive units of Spidroin 2, which are known to be very important in the composition of orb-web-weaving spidroins (MaSp2 and fragelliform silk). According to these molecular characteristics, Spidroin 2 was considered to be an MaSp2-like spidroin for Mygalomorphs [30], which may be related to the spiders’ habitat. Mygalomorphae species use silk to build their nests in trees by holding together the foliage or branches with silk. Inclusions of silk fibers are needed to prevent the full expansion of grass blade as well. Thus, the kind of fibers required for such applications should possess a remarkable combination of mechanical properties, such as high tensile strength, stiffness, and toughness, which are observed for major ampullate silks.

### 2.3. Spider Silk and Silkworm Silk

When it comes to comparison of silkworm silk and spider silk properties and further choice of working material, there are lots of factors to be taken into account. *Bombyx mori* silkworm silk is a unique material that has been historically highly valued for its properties. Silkworm cocoons found their industrial application as basic material for the textile industry thousands of years ago [60]. Fibroin is a main structural component of silkworm silk, which is biocompatible, non-immunogenic, and non-toxic material, while the second concomitant of silkworm silk protein threads sericin has allergenic properties. All possible side effects of fibroin-based matrices and low immunogenic properties are always associated with insufficiently washed sericin [61]. Spiders are well-known suppliers of silk for human needs as well. While using spider web as bandage for stanching bleeding wounds, the ancient Greeks observed extraordinary properties of web material such as high biocompatibility, low immunogenicity and lack of allergic reactions [62]. First scientific studies to reveal biomedical properties of spider silk are dated from 1710, when it was discovered that spider silk web is capable of stemming bleeding in human injuries and supporting tissue regeneration at the same time [63]. According to recent reviews, mechanical strength of spider silk sufficiently surpasses that of silkworms, making it favorable material to broad applications [64] (Table 2). Although domestication of arthropods seems unrealizable due to the cannibalistic and territorial behavior of spiders, the spider silk is still intriguing material to explore and adapt to specific requirements, as there are no other natural or human-created material capable of exhibiting a similar combination of characteristics [65].

### 2.4. Native Spider Silk and Its Recombinant Analogs

There are recombinant analogs of natural spidroins that are synthesized in yeast cells *Pichia pastoris* and *Saccharomyces cerevisiae*, mammalian cells, or by using transgenic animals and plants [66]. Recombinant analogs differ from native materials in lower mechanical strength, which can be slightly increased by various chemical and mechanical methods, e.g., by drawing threads in methanol [67] or by crystallization [68]. Based on recombinant analogs, one may create copolymers with properties different from those of native proteins. Despite recent advances in the recombinant silk production, these methodologies still have several issues. The effective mimicking of complex hierarchical processes of the spider silk production remains a serious challenge. The creation of spider silk fiber is a complicated process, occurring upon changes in ionic composition, temperature, pressure and pH [69] in space and time within the gland. Spiders, as opposed to human-made manufacturing, have been perfecting silk production for millions of years. Every day, web frameworks with multiscale organization-controlled fiber diameter and β-sheet crystal size are created by arthropods [70]. Thus, in the present review, we highlight the processing of native spider silk into task-specific composites.

## 3. Modification of Spider Silk by Inorganic Nanomaterials

**Figure 2:**
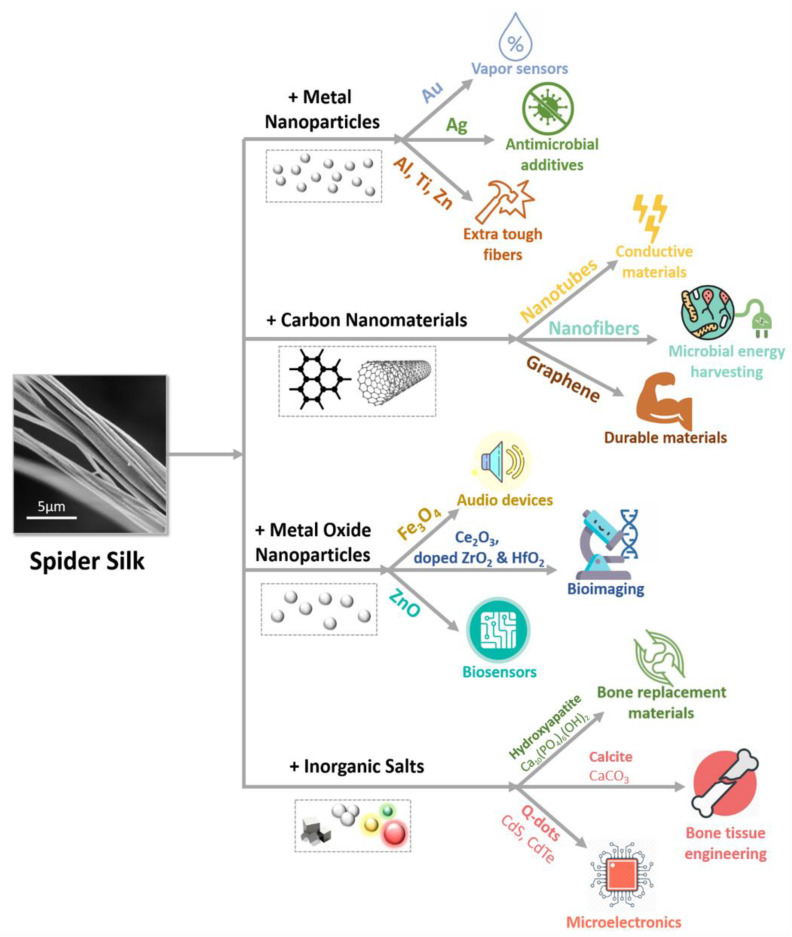
Schematic summary of spider silk modification with inorganic nanomaterials with resulting material applications.

Hybrid materials based on spider silk proteins and inorganic nanomaterials are currently being intensively studied due to their exciting applications in sustainable life technologies, biotechnology, and medicine. Hybrid materials tend to exhibit specific package of properties due to complementing at least two different types of materials. In this context, spider silk provides outstanding mechanical and biocompatible properties while inorganic nanomaterials may offer functional features to harness high-performance hybrid materials (Figure 2).

### 3.1. Metal Nanoparticles

Spider silk fibers have been used as passive templates merely to assemble metal nanoparticles. Mayes et al. showed that spider silk can serve as an excellent template for nanoparticle coating since mechanical properties of the spider silk are not altered consequent to nanoparticle assembly. They were among the first who prepared organic-inorganic hybrid spider silk materials. Although their approach by submerging spider silk filaments in an isopropanol solution of two nm surface-functionalized gold (Au) particles was rather straightforward, the nanoparticle coating was firmly fixed on the fibers resulting in their slightly improved mechanical properties [71]. Later, in contrast, Singh et al. addressed the possibility of employing the spider silk as an active template for blending with metal ions. Moreover, they combined an outstanding physicochemical property of gold nanoparticles, electrical conductivity, with a mechanical supercontracting property of spider silk fibers to produce a novel high-performance material [72]. It is broadly accepted that spider silks contract when exposed to polar environments [73,74] and in case of the gold–silk hybrids, this yields in reduction in the separation between the gold nanoparticles on the fiber surface. Nanoparticle assemblies separated by a dielectric allowed the current flow due to a tunneling process that was shown to be exponentially dependent on the separation between the nanoparticles [75]. Thus, spider silk was used to modulate the electrical conductivity of the gold nanoparticle sheath surrounding spider silk fibers for fabricating a vapor sensor for detecting polar solvents exploiting their polarity [72]. Pertinent to mention is that the other group performed deposition of a thin metallic film of gold nanoparticles to obtain gold-spider silk hybrid fibers, which exhibited decent flexibility for their use as electrodes in microelectronics [76].

A similar approach was used to enhance antimicrobial features of spider silk [77,78] by blending it with silver (Ag) nanoparticles that are known for their exceptional antimicrobial properties [79]. Spider silk hydrolyzed in sodium hydroxide (NaOH) was added to silver nitrate (AgNO_3_) solution for the reduction of silver ions (Ag^+^). During the synthesis of silver nanoparticles, spidroins served as capping and stabilization molecules. The resulting hybrid solutions exhibited not only antimicrobial activities against several multi-drug resistant clinical bacteria [80] but also showed anticoagulant and thrombolytic properties [81]. Such materials can be applied as additives protecting against microbial attack or even as thrombolytic agents and carriers of thrombolytic drugs [82].

In the field of modification of spider silk fibers by metal nanoparticles, a striking example is the use of simple substances to impart superior strength properties to the spider silk. It has been shown that metals can be deliberately incorporated into the inner protein structures of biomaterials through multiple pulsed vapor-phase infiltration. Due to the introduction of metal ions such as zinc (Zn^2+^), titanium (Ti^4+^), or aluminum (Al^3+^) in combination with water from the corresponding precursors into spider silk it became possible to critically improve toughness of the resulting silks [29].

### 3.2. Metal Oxide Nanoparticles

Metal oxide nanoparticles are frequently used as emerging agents for design of the hybrid materials [83]. Consequently, several metal oxides have been used for fabricating spider silk hybrids with advanced properties without significant change of spidroin backbone and, hence, with retained specific spider silk properties. Thus, magnetite (Fe_3_O_4_) nanoparticles were used to provide well-defined and relatively stable silk coatings, possibly due to hydrogen bonding interactions at the oxide-silk interface [70]. Employing magnetic properties of the nanoparticles, such functional hybrid fibers can be integrated into audio devices where strong fabrics responding to a magnetic field are required. For example, a bioinspired hybrid spider silk-based system was developed as an ideal resonator for directional air-sensing across various frequencies, which are often too quiet for traditional microphones to pick up on [84].

As demonstrated by recent research, the use of degummed regenerated (i.e., dissolved) spider silk for preparation of advanced bionanomaterials is of particular interest for biomedical applications [85]. In this context, spider silk fibers regenerated in tetrabutylammonium hydroxide were modified with magnetite (Fe_3_O_4_) nanoparticles. Magnetic hybrid nanofibers showed antibacterial (against both Gram positive and negative) and non-cytotoxic properties (against human lung carcinoma cells) due to biocompatible and antibacterial properties of the spider silk. Meanwhile, the incorporation of magnetic nanoparticles has enhanced the antibacterial efficiency of the fibers [86]. Additionally, peptides of hydrolyzed spider silk, which contain nucleophilic hydroxyls of cysteine, aspartic acid, and amines of histidine, can bind, for example, zinc oxide (ZnO) nanoparticles and bio-assist in the crystal growth of ZnO particles through the aggregation-driven mineralization [87]. Improvement in mechanical characteristics for potential applications in biomaterials were observed via in situ growth of TiO_2_, Al_2_O_3_ in the spider silk via atomic layer deposition (ALD) infiltration [29,88]. More detailed discussion of the influence of oxides on the properties of spider silk is provided in the book of Cohen and Moussian [89]. The development of such bio-mineralized materials coupled with spider silk yields their applications as biosensors for biomolecular detection or optoelectronic nano devices. As a particular example, ceria (Ce_2_O_3_) nanoparticles have been added in situ to recombinant spider silk solution and electrospun into hybrid nanofibers forming mats [90]. The embedded ceria nanoparticles introduced new mechanical and optical properties to the spider silk nanofibers owing to optical properties of trivalent cerium ions, associated with formed oxygen vacancies. The resulting optical fluorescent spider silk nano hybrids could be used in different biomedical or sensing applications [91].

Additionally, metal oxides suit well for the preparation of upconverting luminescent materials as matrices for doping owing to their chemical and photochemical stability, high refractive indices and low phonon energies [92,93]. As such, due to the modification of native spider silk fibers with nanoparticles of zirconia (ZrO_2_) and hafnia (HfO_2_) doped with rare earth elements, upconversion luminescent properties of the nanoparticles were extended to macro-scale components of the fibrous hybrids while the obtained materials remained biocompatible [94]. These optically active hybrid materials based on spider silk and inorganic nanoparticles may find applications in the fields of biosensors and bio-imaging in a noninvasive and real-time manner.

### 3.3. Nanomaterials Based on Inorganic Salts

Inorganic salts are also of great interest for producing hybrid materials. Among them, various phases of calcium carbonates (CaCO_3_) are widespread mineral salts formed in biological systems [95]. As it is the most thermodynamically stable polymorph of calcium carbonate, calcite is a biodegradable material with high biomineralization [96], and is widely used in industry [97]. To extend bio-adaptive properties of calcite to spider silk fibers, Mehta and Hede drop-coated silk with calcium chloride (CaCl_2_) and obtained spider silk-calcite hybrids [98]. Resulting mechanical properties of spider silk complemented inelastic ones of calcite [99]. In the same way, it is possible to bio-mineralize spider silks with osteoconductive hydroxyapatite Ca_10_(PO_4_)_6_(OH)_2_ salts by repetitive cycles of silk incubation in calcium phosphate rich solutions and subsequent washing with water [100]. Both obtained calcite-spider silk hybrids and hydroxyapatite-spider silk hybrids permitted producing new scaffold materials for bone tissue engineering or bone replacement materials [98,100].

The quantum dots are the most widely used fluorescence nanomaterials, which can be used for imparting useful optical properties to hybrid materials. For instance, in different studies, dip-coating with cadmium sulfide (CdS) [71] clusters and layer-by-layer electrostatic absorption of cadmium telluride (CdTe) quantum dots [101] have been used for assembly onto spider silk fibers. The obtained quantum dot-spider silk hybrids exhibited core–shell structure characteristics and emitted bright fluorescence while spider silk mechanical properties were not affected [101]. These fluorescent spider silk-based materials may find applications in microelectronics and biomedicine.

### 3.4. Carbon Nanomaterials

Noteworthy is the use of carbon nanotubes for fabricating tough but lightweight and flexible, multi-functional conductive spider silk hybrid fibers. These can be provided by several approaches that include water-based [102] and dry-coating methods [103] or direct fiber reinforcing during spinning in spider [104] or electrospinning of synthetic silk [105]. It is also interesting to note the work related to simulations of the behavior of spider silk fibers upon the addition of carbon nanotubes [106]. Although spider silk is too simplified in composition there, the model provided can improve the design of novel super tough materials.

Due to presence of both conductive carbon nanotubes and the spider silk within the hybrids, the resulting materials were reversibly affected by strain and humidity, while the charge carrier transport was primarily driven by inter-tube charge hopping [102]. Producing spider silk fibers reinforced by carbon nanotubes and graphene yielded fibers with greatly improved mechanical properties surpassing synthetic polymeric high-performance fibers [104]. Moreover, hybrid carbon nanotube-spider silk fibers could direct the cell growth and simultaneously record signals evoked from cell beating [103]. There also has been published a work related to the formation of hybrid synthetic fibrils with enhanced strength and toughness using electrospinning of synthetic spider silk with carbon nanotubes dispersed in the spinning dope [105]. In addition, carbon nanofibers were synthesized via pyrolysis, using spider silk as precursor, to construct hybrid materials with superior oxygen reduction activity thus providing opportunities in unconventional microbial energy harvesting [107]. As a result, hybrid fibers based on spider silk and carbon nanomaterials can be extensively used as durable custom-shapeable, sensors, and actuating bio-devices. These fibers are also biomedically useful as sutures, surgical implants, and tissue engineering scaffolds due to their enhanced mechanical properties and as electrodes for brain/machine interfaces and neuron regeneration due to the electro conductive properties of the doping agent.

## 4. Trends in Manufacturing Hybrid Silk-Based Materials

Since the trend for silk-based materials synthesis—including the manufacturing of organic-inorganic composite materials—is rapidly developing, the effective production techniques are also under close study and modification. The self-assembly transition of native silk dope into solid fiber in the spider abdomen has inspired researchers to reproduce such mechanism artificially [108]. Several silk design fabrication methods have already been described [109], the most attractive being 3D printing. This approach permits the production of frameworks with complex geometries [110], which are often extremely difficult or impossible to fabricate using traditional techniques. 3D printing is a promising solution to develop materials for biomedical applications in such areas as tissue engineering and regenerative medicine [111,112]. The principles of this printing technique have been studied for widely used bio matrices, for example, gelatin, agar, and alginate. These systems, with the addition of physical or chemical cross-linkers, undergo a liquid-solid transition during printing and transform into hydrogel [113]. Similar processes are possible for printing spider silk-based ink, since spider silk possesses the same proteinaceous structure as widely used bio-composites [114]. Moreover, numerous similarities between natural spinning and the artificial manufacturing of silk derivatives have been realized [114]. This phenomenon can contribute to the production of silk-based frameworks with shapes distinct from those of natural fiber, which is extremely favorable for tunable implant manufacturing.

The presence of inorganic components in the composition of silk-based inks undoubtedly complicates the printing process. For example, the addition of metal particles increases ink density, complicating the sol-gel transition process. However, such difficulties can be resolved by optimizing metal nanocomposite concentration. It should both preserve desired properties of the hybrid and affect insignificantly the printing process. Inclusion of inorganic components in silk ink can potentially solve the issue of low structural integrity and the mechanical performance of printed silk-based materials when compared to native fibers [114]. The treatment of native silk threads with certain inorganic particles has already been proven to positively affect the mechanical properties of hybrid silk fibers [84]. Thus, in the case of silk ink, inorganic components can act as both a necessary support for the protein framework, as well as a source of various additional properties, whether it is increased electrical conductivity, antibacterial properties, or upconversion activity [115].

## 5. Future Directions and Concluding Remarks

The range of applications of the spider silk is very broad due to its unsurpassed biophysicochemical properties and high degree of adaptability. Spider silk provides good basis for formation of the hybrid functional materials with an expanded spectrum of utility.

**Figure 3:**
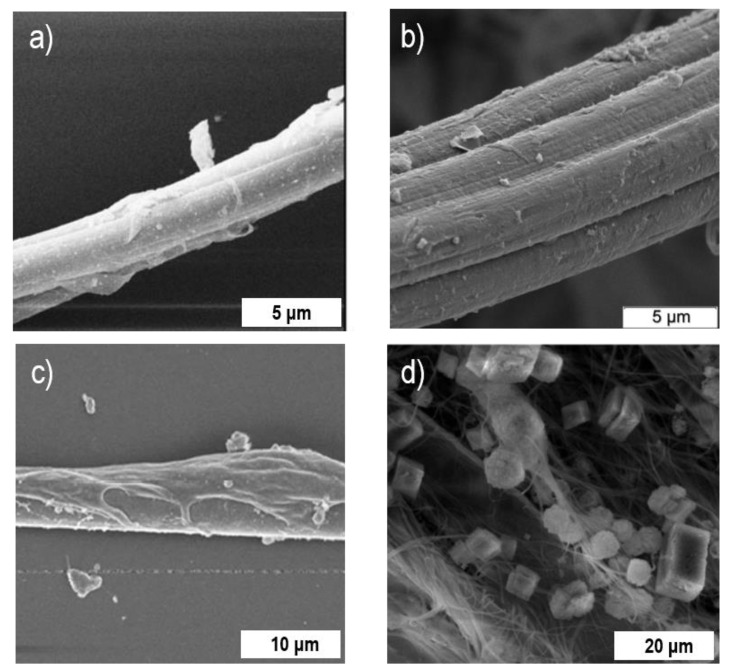
Scanning electron micrographs of spider silk fibers modified with: (**a**) metal nanoparticles (Au) [72], Reproduced with permission from [72]. John Wiley and Sons, 2007. (**b**) metal oxide nanoparticles (ZrO_2_) [84], Reproduced with permission from [84]. PNAS, 2008. (**c**) carbon nanomaterials (carbon nanotubes) [103], Reproduced with permission from [103]. American Chemical Society, 2018. (**d**) inorganic salts (CaCO_3_) [99].

However, based on the limited number of the available studies on developing hybrids based on inorganic nanomaterials and spider silk, it can be concluded that this direction is only at its first stages of development. Moreover, while most of the research activities have been focused on silkworm silk-based functional materials, the limited studies regarding spider silk modifications have thus far revealed fascinating opportunities for new material designs. Most of the methods for producing hybrid materials based on native spider silk and inorganic nanomaterials are straightforward, easy to perform, and environmentally benign, providing opportunity for routinely fabricating spider silk hybrids with different nanomaterials without destroying the fibrous structure regardless of the type of nano objects (Figure 3).

Moreover, mild conditions of its natural biosynthesis may suggest that the formation of derived smart and functional materials will bring about innovative green fabrication processes with a minimal ecological footprint. Therefore, the research concerning spider silk is of high potential in the development of high-performance materials.

Harnessing favorable properties of spider silk and inorganic nanomaterials, existing hybrid formation methods offer a promising direction toward producing structurally and functionally optimized spider silk-based materials. Meanwhile, future work toward understanding the compatibility between spider silk and functional nanomaterials is essential to advance the use of these hybrids in electronic, magnetic, and biomedical applications. Additionally, a better understanding of the key features of forming spider silk-based hybrids will permit developing of the novel protocols that can generate ideas for more affordable and reliable approaches to the production of advanced hybrid materials. One method of efficient hybrid material formation is 3D printing since this process is biomimetic to natural silk spinning and allows the production of materials with complex shapes. Introduction of metal ions as reinforcements in spider silk structure can potentially serve as a model for a more general approach to improving the strength and toughness of other biomaterials and be extended to other biological systems leading to a new class of bioinspired hybrid materials.

## References

[1] Ng L.Y., Mohammad A.W., Leo C.P., Hilal N. (2013). Polymeric membranes incorporated with metal/metal oxide nanoparticles: A comprehensive review. Desalination.

[2] Okada A., Usuki A. (1995). The chemistry of polymer-clay hybrids. Mater. Sci. Eng. C.

[3] Godovski D.Y. (1995). Electron behavior and magnetic properties of polymer nanocomposites. Advances in Polymer Science.

[4] Vollrath F., Porter D. (2009). Silks as ancient models for modern polymers. Polymer.

[5] Holland C., Numata K., Rnjak-Kovacina J., Seib F.P. (2018). The biomedical use of silk: Past, present, future. Adv. Heal. Mater..

[6] Lau A.K.T., Cheung K.H.Y. (2017). Natural Fiber-Reinforced Polymer-Based Composites.

[7] Mohammed L., Ansari M.N.M., Pua G., Jawaid M., Islam M.S. (2015). A review on natural fiber reinforced polymer composite and its applications. Int. J. Polym. Sci..

[8] Saba N., Tahir P.M., Jawaid M. (2014). A review on potentiality of nano filler/natural fiber filled polymer hybrid composites. Polymers.

[9] Porter D., Guan J., Vollrath F. (2012). Spider silk: Super material or thin fibre?. Adv. Mater..

[10] Singh K., Maity S., Singha M. (2012). Spinning and applications of spider silk. Front. Sci..

[11] Vollrath F. (2000). Strength and structure of spiders’ silks. Rev. Mol. Biotechnol..

[12] Shao Z., Vollrath F. (2002). Surprising strength of silkworm silk. Nature.

[13] Du N., Yang Z., Liu X.Y., Li Y., Xu H.Y. (2010). Structural origin of the strain-hardening of spider silk. Adv. Funct. Mater..

[14] Allmeling C., Jokuszies A., Reimers K., Kall S., Vogt P.M. (2007). Use of spider silk fibres as an innovative material in a biocompatible artificial nerve conduit. J. Cell. Mol. Med..

[15] Hakimi O., Knight D.P., Vollrath F., Vadgama P. (2007). Spider and mulberry silkworm silks as compatible biomaterials. Compos. Part B Eng..

[16] Vepari C., Kaplan D.L. (2007). Silk as a biomaterial. Prog. Polym. Sci..

[17] Yarger J.L., Cherry B.R., van der Vaart A. (2018). Uncovering the structure-function relationship in spider silk. Nat. Rev. Mater..

[18] Zhang Y., Zhao A.C., Sima Y.H., Lu C., Xiang Z.H., Nakagaki M. (2013). The Molecular structures of major ampullate silk proteins of the wasp spider, argiope bruennichi: A second blueprint for synthesizing de novo silk. Comp. Biochem. Physiol. B Biochem. Mol. Biol..

[19] Lin Z., Huang W., Zhang J., Fan J.S., Yang D. (2009). Solution structure of eggcase silk protein and its implications for silk fiber formation. Proc. Natl. Acad. Sci. USA.

[20] Jin H.J., Kaplan D.L. (2003). Mechanism of silk processing in insects and spiders. Nature.

[21] Hu X., Vasanthavada K., Kohler K., McNary S., Moore A.M.F., Vierra C.A. (2006). Molecular mechanisms of spider silk. Cell. Mol. Life Sci..

[22] Dicko C., Vollrath F., Kenney J.M. (2004). Spider silk protein refolding is controlled by changing PH. Biomacromolecules.

[23] Hedhammar M., Rising A., Grip S., Martinez A.S., Nordling K., Casals C., Stark M., Johansson J. (2008). Structural properties of recombinant nonrepetitive and repetitive parts of major ampullate Spidroin 1 from Euprosthenops Australis: Implications for fiber formation. Biochemistry.

[24] Kluge J.A., Rabotyagova O., Leisk G.G., Kaplan D.L. (2008). Spider silks and their applications. Trends Biotechnol..

[25] Reches M., Gazit E. (2006). Controlled patterning of aligned self-assembled peptide nanotubes. Nat. Nanotechnol..

[26] Knowles T.P.J., Oppenheim T.W., Buell A.K., Chirgadze D.Y., Welland M.E. (2010). Nanostructured films from hierarchical self-assembly of amyloidogenic proteins. Nat. Nanotechnol..

[27] Rominger K.M., Nestor G., Eriksson J.E., Seisenbaeva G.A., Kessler V.G. (2019). Complexes of Keggin POMs [PM_12_O_40_]^3–^ (M = Mo, W) with GlyGly Peptide and Arginine—Crystal structures and solution reactivity. Eur. J. Inorg. Chem..

[28] Kiseleva A.P., Krivoshapkin P.V., Krivoshapkina E.F. (2020). Recent advances in development of functional spider silk-based hybrid materials. Front. Chem..

[29] Lee S.M., Pippel E., Gösele U., Dresbach C., Qin Y., Chandran C.V., Bräuniger T., Hause G., Knez M. (2009). Greatly increased toughness of infiltrated spider silk. Science.

[30] Huang W., Ling S., Li C., Omenetto F.G., Kaplan D.L. (2018). Silkworm silk-based materials and devices generated using bio-nanotechnology. Chem. Soc. Rev..

[31] Bittencourt D., Dittmar K., Lewis R.V., Rech E.L. (2010). A MaSp2-like gene found in the Amazon mygalomorph spider Avicularia juruensis. Comp. Biochem. Physiol. Part B Biochem. Mol. Biol..

[32] Vehoff T., Glišović A., Schollmeyer H., Zippelius A., Salditt T. (2007). Mechanical properties of spider dragline silk: Humidity, hysteresis, and relaxation. Biophys. J..

[33] Barghout J.Y., Thiel B.L., Viney C. (1999). Spider (Araneus diadematus) cocoon silk: A case of non-periodic lattice crystals with a twist?. Int. J. Biol. Macromol..

[34] Brooks A.E., Stricker S.M., Joshi S.B., Kamerzell T.J., Middaugh C.R., Lewis R.V. (2008). Properties of synthetic spider silk fibers based on Argiope aurantia MaSp2. Biomacromolecules.

[35] Lewis R.V., Hinman M., Kothakota S., Fournier M.J. (1996). Expression and purification of a spider silk protein: A new strategy for producing repetitive proteins. Protein Expr. Purif..

[36] Simmons A., Ray E., Jelinski L.W. (1994). Solid-state 13C NMR of Nephila clavipes dragline silk establishes structure and identity of crystalline regions. Macromolecules.

[37] Parkhe A.D., Seeley S.K., Gardner K., Thompson L., Lewis R.V. (1997). Structural studies of spider silk proteins in the fiber. J. Mol. Recognit..

[38] Hayashi C.Y., Shipley N.H., Lewis R.V. (1999). Hypotheses that correlate the sequence, structure, and mechanical properties of spider silk proteins. Int. J. Biol. Macromol..

[39] Bram A., Bränden C., Craig C., Snigireva I., Riekel C. (1997). X-ray diffraction from single fibres of spider silk. J. Appl. Crystallogr..

[40] Scheibel T. (2004). Spider silks: Recombinant synthesis, assembly, spinning, and engineering of synthetic proteins. Microb. Cell Factories.

[41] Huemmerich D., Helsen C.W., Quedzuweit S., Oschmann J., Rudolph R., Scheibel T. (2004). Primary structure elements of spider dragline silks and their contribution to protein solubility. Biochemistry.

[42] Hinman M.B., Jones J.A., Lewis R.V. (2000). Synthetic spider silk: A modular fiber. Trends Biotechnol..

[43] Rising A., Nimmervoll H., Grip S., Fernandez-Arias A., Storckenfeldt E., Knight D.P., Vollrath F., Engström W. (2005). Spider silk proteins—Mechanical property and gene sequence. Zool. Sci..

[44] Hayashi C.Y., Lewis R.V. (1998). Evidence from flagelliform silk cDNA for the structural basis of elasticity and modular nature of spider silks. J. Mol. Biol..

[45] Casem M.L., Collin M.A., Ayoub N.A., Hayashi C.Y. (2010). Silk gene transcripts in the developing tubuliform glands of the Western black widow, Latrodectus hesperus. J. Arachnol..

[46] Blasingame E., Tuton-Blasingame T., Larkin L., Falick A.M., Zhao L., Fong J., Vaidyanathan V., Visperas A., Geurts P., Hu X. (2009). PyriformSpidroin 1, a novel member of the silk gene family that anchors dragline silk fibers inattachment discs of the black widow spider, Latrodectus hesperus. J. Biol. Chem..

[47] Hayashi C.Y., Blackledge T.A., Lewis R.V. (2004). Molecular and mechanical characterization of acini-form silk: Uniformity of iterated sequence modules in a novel member of the spider silk fibroingene family. Mol. Biol. Evol..

[48] Agnarsson I., Blackledge T.A. (2009). Can a spider web be too sticky? Tensile mechanics constrains the evolution of capture spiral stickiness in orb-weaving spiders. J. Zool..

[49] Kerr G.G., Nahrung H.F., Wiegand A., Kristoffersen J., Killen P., Brown C., Macdonald J. (2018). Mechanical properties of silk of the Australian golden orb weavers Nephila pilipes and Nephila plumipes. Biol. Open.

[50] Vienneau-Hathaway J.M., Brassfield E.R., Lane A.K., Collin M.A., Correa-Garhwal S.M., Clarke T.H., Schwager E.E., Garb J.E., Hayashi C.Y., Ayoub N.A. (2017). Duplication and concerted evolution of MiSp-encoding genes underlie the material properties of minor ampullate silks of cobweb weaving spiders. BMC Evol. Biol..

[51] Perea G.B., Riekel C., Guinea G.V., Madurga R., Daza R., Burghammer M., Hayashi C., Elices M., Plaza G.R., Pérez-Rigueiro J. (2013). Identification and dynamics of polyglycine II nanocrystals in Argiope trifasciata flagelliform silk. Sci. Rep..

[52] Van Nimmen E., Gellynck K., Gheysens T., Van Langenhove L., Mertens J. (2005). Modeling of the stress-strain behavior of egg sac silk of the spider Araneus diadematus. J. Arachnol..

[53] Greco G., Wolff J.O., Pugno N.M. (2020). Strong and tough silk for resilient attachment discs: The mechanical properties of piriform silk in the Spider Cupiennius salei (Keyserling, 1877). Front. Mater..

[54] Blackledge T.A., Hayashi C.Y. (2006). Unraveling the mechanical properties of composite silk threads spun by cribellate orb-weaving spiders. J. Exp. Biol..

[55] Coyle F.A., Shear W.A. (1986). The role of silk in prey capture by Nonaraneomorph spiders. Spiders: Webs, Behavior and Evolution.

[56] Palmer J.M. (1985). The silk and silk production system of the funnel-web mygalomorph spider Euagrus (Araneae, Diplurudae). J. Morphol..

[57] Hajer J., Karschová S., Řeháková D. (2016). Silks and silk-producing organs of Neotropical tarantula Avicularia metallica (Araneae, Mygalomorphae, Theraphosidae). Ecol. Montenegrina.

[58] Garb J.E., diMauro T., Lewis R.V., Hayashi C.Y. (2007). Expansion and intragenic homogenization of spider silk genes since the Triassic: Evidence from Mygalomorphae (tarantulas and their kin) spidroins. Mol. Biol. Evol..

[59] Collin M.A., Garb J.E., Edgerly J.S., Hayashi C.Y. (2009). Characterization of silk spun by the embiopteran, *Antipaluria urichi*. Insect Biochem. Mol. Biol..

[60] Malay A.D., Sato R., Yazawa K., Watanabe H., Ifuku N., Masunaga H., Numata K., Hikima T., Guan J., Mandal B.B. (2016). Relationships between physical properties and sequence in silkworm silks. Sci. Rep..

[61] Chirila T.V., Suzuki S., Bray L.J., Barnett N.L., Harkin D.G. (2013). Evaluation of silk sericin as a biomaterial: In vitro growth of human corneal limbal epithelial cells on Bombyx mori sericin membranes. Prog. Biomater..

[62] Altman G.H., Diaz F., Jakuba C., Calabro T., Horan R.L., Chen J., Kaplan D.L. (2003). Silk-based biomaterials. Biomaterials.

[63] Bon F.X. (1710). A discourse upon the usefulness of the silk of spiders. By Monsieur Bon, President of the Court of Accounts, Aydes and Finances, and President of the Royal Society of Science at Montpellier. Communicated by author. Philos. Trans. R. Soc. Lond..

[64] Andersson M., Johansson J., Rising A. (2016). Silk spinning in silkworms and spiders. Int. J. Mol. Sci..

[65] Heim M., Keerl D., Scheibel T. (2009). Spider silk: From soluble protein to extraordinary fiber. Angew. Chem..

[66] Yang J., Barr L.A., Fahnestock S.R., Liu Z.-B. (2005). High yield recombinant silk- like protein production in transgenic plants through protein targeting. Transgenic Resour..

[67] Slotta U., Tammer M., Kremer F., Koelsch P., Scheibel T. (2006). Structural analysis of spider silk films. Supramol. Chem..

[68] Lazaris A., Arcidiacono S., Huang Y., Zhou J.F., Duguay F., Chretien N., Welsh E.A., Soares J.W., Karatzas C.N. (2002). Spider silk fibers spun from soluble recombinant silk produced in mammalian cells. Science.

[69] Foo C.W.P., Bini E., Hensman J., Knight D.P., Lewis R.V., Kaplan D.L. (2006). Role of pH and charge on silk protein assembly in insects and spiders. Appl. Phys. A.

[70] Buehler M.J. (2013). Materials by design—A perspective from atoms to structures. MRS Bull..

[71] Mayes E.L., Vollrath F., Mann S.C. (1998). Fabrication of magnetic spider silk and other silk-fiber composites using inorganic nanoparticles. Adv. Mater..

[72] Singh A., Hede S., Sastry M. (2007). Spider silk as an active scaffold in the assembly of gold nanoparticles and application of the gold—silk bioconjugate in vapor sensing. Small.

[73] Boutry C., Blackledge T.A. (2010). Evolution of supercontraction in spider silk: Structure-function relationship from tarantulas to orb-weavers. J. Exp. Biol..

[74] Shao Z., Vollrath F. (1999). The effect of solvents on the contraction and mechanical properties of spider silk. Polymer.

[75] Krasteva N., Besnard I., Guse B., Bauer R.E., Müllen K., Yasuda A., Vossmeyer T. (2002). Self-assembled gold nanoparticle/dendrimer composite films for vapor sensing applications. Nano Lett..

[76] Steven E., Park J.G., Paravastu A., Lopes E.B., Brooks J.S., Englander O., Siegrist T., Kaner P., Alamo R.G. (2011). Physical characterization of functionalized spider silk: Electronic and sensing properties. Sci. Technol. Adv. Mater..

[77] Chakraborty D., Das S. (2009). Antibacterial activities of cobweb protein. Clin. Microbiol. Infect..

[78] Roozbahani H., Asmar M., Ghaemi N., Issazadeh K. (2014). Evaluation of antimicrobial activity of spider silk pholcus phalangioides against two bacterial pathogens in food borne. Int. J. Adv. Biol. Biomed. Res..

[79] Kim J.S., Kuk E., Yu K.N., Kim J.H., Park S.J., Lee H.J., Kim S.H., Park Y.K., Park Y.H., Hwang C.Y. (2007). Antimicrobial Effects of Silver Nanoparticles. Nanomed. Nanotechnol. Biol. Med..

[80] Lateef A., Ojo S.A., Azeez M.A., Asafa T.B., Yekeen T.A., Akinboro A., Oladipo I.C., Gueguim-Kana E.B., Beukes L.S. (2016). Cobweb as novel biomaterial for the green and eco-friendly synthesis of silver nanoparticles. Appl. Nanosci..

[81] Lateef A., Ojo S.A., Elegbede J.A., Azeez M.A., Yekeen T.A., Akinboro A. (2017). Evaluation of some biosynthesized silver nanoparticles for biomedical applications: Hydrogen peroxide scavenging, anticoagulant and thrombolytic activities. J. Clust. Sci..

[82] McCarthy J.R., Sazonova I.Y., Erdem S.S., Hara T., Thompson B.D., Patel P., Botnaru I., Lin C.P., Reed G.L., Weissleder R. (2012). Multifunctional nanoagent for thrombus-targeted fibrinolytic therapy. Nanomedicine.

[83] Oskam G. (2006). Metal oxide nanoparticles: Synthesis, characterization and application. J. Sol Gel Sci. Technol..

[84] Zhou J., Miles R.N. (2017). Sensing fluctuating airflow with spider silk. Proc. Natl. Acad. Sci. USA.

[85] Seidel A., Liivak O., Calve S., Adaska J., Ji G., Yang Z., Grubb D., Zax D.B., Jelinski L.W. (2000). Regenerated spider silk:  Processing, properties, and structure. Macromolecules.

[86] Singh N., Mondal D., Sharma M., Devkar R.V., Dubey S., Prasad K. (2015). Sustainable processing and synthesis of nontoxic and antibacterial magnetic nanocomposite from spider silk in neoteric solvents. ACS Sustain. Chem. Eng..

[87] Huang Z., Yan D., Yang M., Liao X., Kang Y., Yin G., Yao Y., Hao B. (2008). Preparation and characterization of the biomineralized zinc oxide particles in spider silk peptides. J. Colloid Interface Sci..

[88] Lee S.M., Pippel E., Moutanabbir O., Kim J.H., Lee H.J., Knez M. (2014). In situ raman spectroscopic study of al-infiltrated spider dragline silk under tensile deformation. ACS Appl. Mater. Interfaces.

[89] Cohen E., Moussian B. (2016). Extracellular Composite Matrices in Arthropods.

[90] Kandas I., Shehata N., Hassounah I., Sobolčiak P. (2018). Optical fluorescent spider silk electrospun nanofibers with embedded cerium oxide nanoparticles. J. Nanophotonics.

[91] Tow K.H., Chow D.M., Vollrath F., Dicaire I., Gheysens T., Thévenaz L. (2018). Exploring the use of native spider silk as an optical fiber for chemical sensing. J. Lightwave Technol..

[92] Auzel F. (2004). Upconversion and anti-stokes processes with f and d ions in solids. Chem. Rev..

[93] Chatterjee D.K., Rufaihah A.J., Zhang Y. (2008). Upconversion fluorescence imaging of cells and small animals using lanthanide doped nanocrystals. Biomaterials.

[94] Kiseleva A., Kiselev G., Kessler V., Seisenbaeva G., Gets D., Rumyantseva V., Lyalina T., Fakhardo A., Krivoshapkin P., Krivoshapkina E. (2019). Optically active hybrid materials based on natural spider silk. ACS Appl. Mater. Interfaces.

[95] Berman A., Hanson J., Leiserowitz L., Koetzle T.F., Weiner S., Addadi L. (1993). Biological control of crystal texture: A widespread strategy for adapting crystal properties to function. Science.

[96] Xu X., Han J.T., Cho K. (2004). Formation of amorphous calcium carbonate thin films and their role in biomineralization. Chem. Mater..

[97] Montes-Hernandez G., Fernández-Martínez A., Charlet L., Tisserand D., Renard F. (2008). Textural properties of synthetic nano-calcite produced by hydrothermal carbonation of calcium hydroxide. J. Cryst. Growth.

[98] Mehta N., Hede S. (2005). Spider silk calcite composite. Hypothesis.

[99] Dmitrović S., Jokić B., Prekajski M., Pantić J., Zmejkoski D., Zarubica A., Matović B. (2016). Synthesis and characterization of spider silk calcite composite. Process. Appl. Ceram..

[100] Cao B., Mao C. (2007). Oriented nucleation of hydroxylapatite crystals on spider dragline silks. Langmuir.

[101] Chu M., Sun Y. (2007). Self-Assembly method for the preparation of near-infrared fluorescent spider silk coated with cdte nanocrystals. Smart Mater. Struct..

[102] Steven E., Saleh W.R., Lebedev V., Acquah S.F.A., Laukhin V., Alamo R.G., Brooks J.S. (2013). Carbon nanotubes on a spider silk scaffold. Nat. Commun..

[103] Hou J., Xie Y., Ji A., Cao A., Fang Y., Shi E. (2018). Carbon-nanotube-wrapped spider silks for directed cardiomyocyte growth and electrophysiological detection. ACS Appl. Mater. Interfaces.

[104] Lepore E., Bosia F., Bonaccorso F., Bruna M., Taioli S., Garberoglio G., Ferrari A.C., Pugno N.M. (2017). Spider silk reinforced by graphene or carbon nanotubes. 2D Mater..

[105] Firm T.H.E., Karlf O.F. (2007). Method for Separating Trivalent Americium from Trivalent Curium. U.S. Patent.

[106] Bosia F., Buehler M.J., Pugno N.M. (2010). Hierarchical simulations for the design of supertough nanofibers inspired by spider silk. Phys. Rev. E.

[107] Zhou L., Fu P., Cai X., Zhou S., Yuan Y. (2016). Naturally derived carbon nanofibers as sustainable electrocatalysts for microbial energy harvesting: A new application of spider silk. Appl. Catal. B Environ..

[108] Porter D., Vollrath F. (2009). Silk as a biomimetic ideal for structural polymers. Adv. Mater..

[109] Wu Y., Shah D.U., Liu C., Yu Z., Liu J., Ren X., Rowland M.J., Abell C., Ramage M.H., Scherman O.A. (2017). Bioinspired supramolecular fibers drawn from a multiphase self-assembled hydrogel. Proc. Natl. Acad. Sci. USA.

[110] Truby R.L., Lewis J.A. (2016). Printing soft matter in three dimensions. Nature.

[111] Jammalamadaka U., Tappa K. (2018). Recent advances in biomaterials for 3D printing and tissue engineering. J. Funct. Biomater..

[112] Gopinathan J., Noh I. (2018). Recent trends in bioinks for 3D printing. Biomater. Res..

[113] Sankaran S., Zhao S., Muth C., Paez J., del Campo A. (2018). Toward light-regulated living biomaterials. Adv. Sci..

[114] Mu X., Fitzpatrick V., Kaplan D. (2020). From silk spinning to 3D printing: Polymer manufacturing using directed hierarchical molecular assembly. Adv. Healthc. Mater..

[115] DeSimone E., Jungst T., Schacht K., Groll J., Scheibel T. (2015). Biofabrication of 3D constructs: Fabrication technologies and spider silk proteins as bioinks. Pure Appl. Chem..

